# Extra-Curricular Activities and Well-Being: Results From a Survey of Undergraduate University Students During COVID-19 Lockdown Restrictions

**DOI:** 10.3389/fpsyg.2021.647402

**Published:** 2021-06-28

**Authors:** Rachael Finnerty, Sara A. Marshall, Constance Imbault, Laurel J. Trainor

**Affiliations:** ^1^Department of Psychology, Neuroscience & Behaviour, McMaster University, Hamilton, ON, Canada; ^2^McMaster Institute for Music and the Mind, Faculty of Science, McMaster University, Hamilton, ON, Canada; ^3^Rotman Research Institute, Baycrest Hospital, Toronto, ON, Canada

**Keywords:** music, wellness, undergraduate students, COVID-19 pandemic, extra-curricular activities, anxiety, stress, mental health

## Abstract

Participation in extra-curricular activities has been found to associate with increased well-being. Here we investigated in a survey (*n* = 786) what activities university students at a Canadian university engaged in during the stressful COVID-19 pandemic lockdown in April, 2020, which coincided with a novel online exam period, and how these activities related to perceived well-being, anxiety (STAI-S), social aspects of activities, and personality. Sixty-five percentage of students scored in the high anxiety category of the STAI-S, an alarming statistic given that only 24% had reached out for professional supports. This is consistent with reports that current supports on university campuses are inadequate. Listening to music (92%) and watching movies/series (92%) were engaged in most frequently, followed by socializing virtually (89%) and engaging in social media (85%). The activities students rated as most helpful to their well-being were somewhat different, with outdoor exercise rated highest, followed by socializing virtually and listening to music. While all activities were rated as beneficial, those with a social component tended to have high ratings, consistent with students attempting to replace lost social interactions. Linear regression models found few associations between STAI-S scores and other measures, likely because of large individual differences and lack of a pre-pandemic baseline needed to assess changes in anxiety. The importance of individual differences was evident in that those higher in *conscientiousness* or *extraversion* or *emotional stability* were more likely to engage in exercise, while those higher in *openness to experience* were more likely to engage in journaling, playing a musical instrument, or singing, with a trend for higher engagement in song writing. Individual differences were also evident in that equal numbers of students gave positive and negative comments related to their well-being during the pandemic. The individual differences uncovered here suggest that having a variety of proactive interventions would likely reach more students. Indeed, 52% indicated an interest in online group music therapy, 48% in art therapy and 40% in verbal therapy, despite music and art therapies being virtually non-existent on campuses. In sum, the findings highlight the importance of choice in extra-curricular activities and therapies that support well-being.

## Introduction

Undergraduate university students fall into an age range that is vulnerable for poor mental health, with significantly higher rates of mental health struggles than the general population (Lee and Jung, 2018). The situation of a global pandemic would be expected to further exacerbate anxiety and impact wellness. In March, 2020, university students in Ontario, Canada were asked to stay home and to self-isolate as a precautionary measure in response to the COVID-19 global pandemic. This timing coincided with the last weeks of classes before final exams, with all classes immediately transitioning to online formats. In addition to the academic disruption, students' in-person social activities were also severely constrained. Despite the transition to isolation, students may have continued participating in extra-curricular activities from home. Extra-curricular activities have been shown to be an important part of facilitating problem-solving, enabling expression of emotion, enhancing adaptability, and contributing to the development of interpersonal skills (Fares et al., 2016). They also appear to be related to the development of self-regulatory mechanisms underpinning psychological and social outcomes, which is of particular importance to this age group as they transition into adult roles (Guilmette et al., 2019).

The goal of the present exploratory research was to investigate participation in, and the impact of, extra-curricular activities on wellness in university students during a time of high stress and social isolation by making use of the convergence of COVID-19 stay-at-home precautionary measures and an exam period that had transitioned to an online format. Specifically, we investigated (1) which extra-curricular activities undergraduate students engaged in on their own volition during this time, (2) which activities students reported as contributing to their wellness, (3) how anxious students were during this time as revealed by scores on a standardized measure of anxiety, (4) relations between anxiety scores and participation in particular activities, (5) whether social aspects of activities impacted anxiety levels, (6**)** whether students' ratings of how key activities affected their well-being related to their anxiety levels, (7) whether personality traits played a role in the activities chosen and on anxiety, (8) how much interest students had in participating in online group music, art and verbal therapy, and (9) how students described their experiences during COVID-19 precautionary measures in an open-ended format.

A number of extra-curricular activities have been studied in relation to wellness and anxiety, including exercise, use of social media, and creative activities, such as music and art. We were particularly interested in music, as it has a long history of supporting humankind through pandemics (Bassler, 2020). Media content today suggests this is also the case in the current pandemic; indeed, a new category of music has been identified—“pandemic pop” (Cross, 2020; Rosen, 2020; Wilson, 2020)—highlighting the importance of music during this time.

Exercise is widely recognized as contributing to health and wellness. Systematic reviews and meta-analyses support that engaging in physical activity/exercise increases wellness (Reed and Buck, 2009; Rodriguez-Ayllon et al., 2019), while recognizing that the amount of exercise is likely important (there may be a minimum amount of weekly exercise needed to impact mental well-being). Although Rodriguez-Ayllon et al. (2019) found that physical activity improved well-being across many different measures, the association between physical activity and anxiety was less clear. Studies examining the impact of sedentary behavior find the expected inverse relationship with anxiety, that is, sedentary behavior has a small, positive association with anxiety (Allen et al., 2019). There are also likely individual differences in the association between exercise and well-being, with the positive effect of engaging in exercise potentially mediated by genetic factors (De Moor et al., 2008).

The impact of social media on wellness and anxiety is controversial. In a sample of adolescents, Woods and Scott (2016) found that greater overall social media use was related to higher levels of anxiety and, in a systemic review, Keles et al. (2020) noted a general association between social media use and mental health struggles in adolescents, although they concluded that the relationship between the two is complex. With regard to sedentary screen time, a meta-analysis of observational studies specific to children and adolescents found that ~1 h a day of screen time associated with decreased symptoms of depression, whereas 2 h or more per day associated with increased symptoms of depression (Liu et al., 2016). It should be noted, however, that these findings were only significant for those younger than 14 years of age. A systematic review and meta-analysis by Wang et al. (2019), also supports that increased screen time results in increased symptoms of depression, although in this analysis the findings were only significant in females. Passive screen time also has been associated with anxiety severity (Maras et al., 2015). The type of screen-based activity, and particularly whether it involves passive vs. active engagement, likely affects whether the impact is positive, negative, or neutral. However, the picture here is far from clear. Video games, but not television or general computer use, may be related to more severe symptoms of anxiety (Maras et al., 2015), but other studies have suggested that video games could have a positive impact on wellness (Johnson et al., 2013).

Creative activities, including music, can reduce anxiety according to Toyoshima et al. (2011). This study found that when people participated in creative arts activities that they had prior experience with, all the activities, including calligraphy, clay molding, and piano playing, reduced anxiety compared to a control group who sat silently for an equivalent amount of time. This suggests that there are likely individual differences in how beneficial a particular creative activity may be. Perruzza and Kinsella (2010) completed a literature review to examine the perceived outcomes of creative arts occupations with respect to health and well-being and concluded that the creative arts support overall wellness. A systematic review of the use of creative arts activities as interventions to promote mental well-being found that nine articles out of 11 highlighted “significant improvements in well-being,” including ratings of mood, self-esteem, and interactions with others (Leckey, 2011). A review of the literature supports the use of music listening to positively impact preoperative anxiety, and more generally to promote relaxation (Biley, 2000). Some studies report that everyday listening to music and creating music may serve as proactive tools to promote or maintain well-being. Linnemann et al. (2015) found that university students had lower subjective stress levels after listening to music in a naturalistic study where students completed assessments multiple times a day over 5 days. Participating in music has also been linked to increased wellness, lowered burnout rates, and is highly recommended during medical training (Fares et al., 2016).

In the present study, to examine the nine questions outlined above, we created an online survey and administered it about 1 month after the start of the COVID-19 precautionary measures, which also coincided with students' exam period, thus creating a situation of potentially high anxiety. To examine the extent of students' participation in different extra-curricular activities, we asked students which activities from a list of 16 they were engaging in (as well as to indicate other activities not listed). For those activities they were engaging in, we asked how much they felt each activity contributed to their well-being and whether they engaged in the activity alone or with others (either in person or virtually). We also obtained a measure of their current state anxiety using the State portion of the standardized State Trait Anxiety Inventory (STAI-S; Spielberger et al., 1983) and a measure of the Big Five personality traits using the standardized Ten-Item Personality Inventory (TIPI; Gosling et al., 2003). Finally, we probed their interest in participating in online group music, art and verbal therapies and asked about their experiences during the COVID-19 precautionary measures in an open-ended question.

To address our nine main questions, we used a combination of descriptive statistics; exploratory linear regression models to determine what factors predicted anxiety scores; and content analysis of the open-ended questions about COVID-19 experiences. Unfortunately, it was not possible to obtain a measure of their state anxiety prior to this stressful period so we were unable to examine how much state anxiety changed from before to during the COVID-19 restrictions. We expected music, exercise, social media and creative arts to be popular activities during the time of the survey and for participation in these activities to correlate with perceived wellness benefit and with STAI-S anxiety scores. We also expected student self-reported ratings of how much an activity contributed to their wellness to be related to their anxiety scores.

Recognizing that social engagement can contribute to wellness (Caldwell et al., 1992; Brajša-Žganec et al., 2011; Doerksen et al., 2014), and that a lack of social connection is associated with poorer health, including increased depression and social anxiety (Morina et al., 2020), we asked students whether they participated in their extra-curricular activities alone or with others. We expected the COVID-19 social restrictions to motivate students to engage in activities that promote a feeling of being connected to others. Additionally, we anticipated that engaging in activities with others, whether in person or virtual, would relate to lower anxiety scores.

Individual differences in personality is a key factor in how different people manage state-anxiety and the enjoyment of extra-curricular activities. A metasynthesis conducted by Strickhouser et al. (2017) suggested that personality predicts overall health and well-being. In addition, several studies indicate that personality predicts which activities people choose to engage in (Wolfradt and Pretz, 2001; Aaron et al., 2011; Gil De Zuniga et al., 2017; Gjermunds et al., 2020). Thus, we investigated how personality, as measured by the TIPI, related to anxiety levels and which extra-curricular activities individuals chose to participate in during the pandemic. As per Bunevicius et al. (2008), we expected the trait of high emotional stability as measured by the TIPI to relate to lower anxiety scores on the STAI-S. We also expected that students who scored higher on the openness trait on the TIPI would engage more in creative activities such as music and art (Kaufman et al., 2016; Gjermunds et al., 2020).

Finally, an important long-term goal of the present study was to inform development of further supports for wellness on university campuses. Currently, in Canada mental health supports are almost entirely limited to verbal therapies and are often only accessible once a crisis has been reached. In thinking about establishing *proactive* supports to help students manage stress and anxiety before they reach critical levels, and while recognizing the importance of individual differences by offering a variety of programs, in the present survey we asked students to indicate their interest in participating in online group music, art, and verbal therapies. Although music and art therapy are used in other situations as interventions for mental and physical conditions, including depression, they are seldom offered on university campuses in Canada. A critical review of art-based therapies in mental health recovery indicates that such interventions can lead to improved self-esteem, self-discovery, empowerment, self-expression, rebuilding of identity, self-validation, motivation, and sense of purpose (Van Lith et al., 2013). In addition to gauging interest in proactive group music, art, and verbal therapies, our content analysis of students' responses to the open-ended question regarding their experiences during the COVID-19 restrictions may further inform the need for, and types of, interventions that would be impactful.

## Methods

### Participants

A convenience sample of 786 full-time undergraduate students at McMaster University completed the survey (144 male, 634 female, 4 non-binary and 4 preferred not to answer; 50% selected the age category 19–20 years; 17% <19 years; 29% 21–22 years; 3% 23–24 years; 1% >24 years). 26 (3%) were international students; the majority (82%) were enrolled in the Faculty of Science; and the majority were living in a detached home (61%) with their parents (69%) and siblings (56%) during the lockdown. 17% reported a psychological or neurological disorder. The TIPI revealed distributions for each of the 10 indicators that closely matched those previously reported for the same age range (Gosling et al., 2003). Questions related to musical background revealed 55% of the students could play an instrument, 6% played an instrument professionally, 44% had an immediate family member who played an instrument, and 26% could play by ear. 41% of students listened to music 1–10 h/week, 29% listened 10–20 h/week, 15% listened 20–30 h/week, and 12% listened more than 30 h/week. When asked to rate how closely they pay attention when listening to music from 1 (music is always background only) to 5 (always pay attention to music), the average rating was 3.31. The top three styles of music listened to were Pop (84%), R&B (63%), and Rap (52%). An additional 126 students began but did not complete the survey. Recruitment methods included social media platforms, direct emails (from Faculty of Science; School of the Arts), and announcements on McMaster's online learning platform Avenue to Learn. We did not have a target number of participants but simply analyzed all completed surveys. It is possible that the sample was biased, as students concerned about their mental health may have been more or less likely to participate. Students were informed that they could win one of twenty $50 CAD prizes by participating in the survey. The protocol was approved by the McMaster Research Ethics Board.

### Materials

#### The Survey

**Figure 1:**
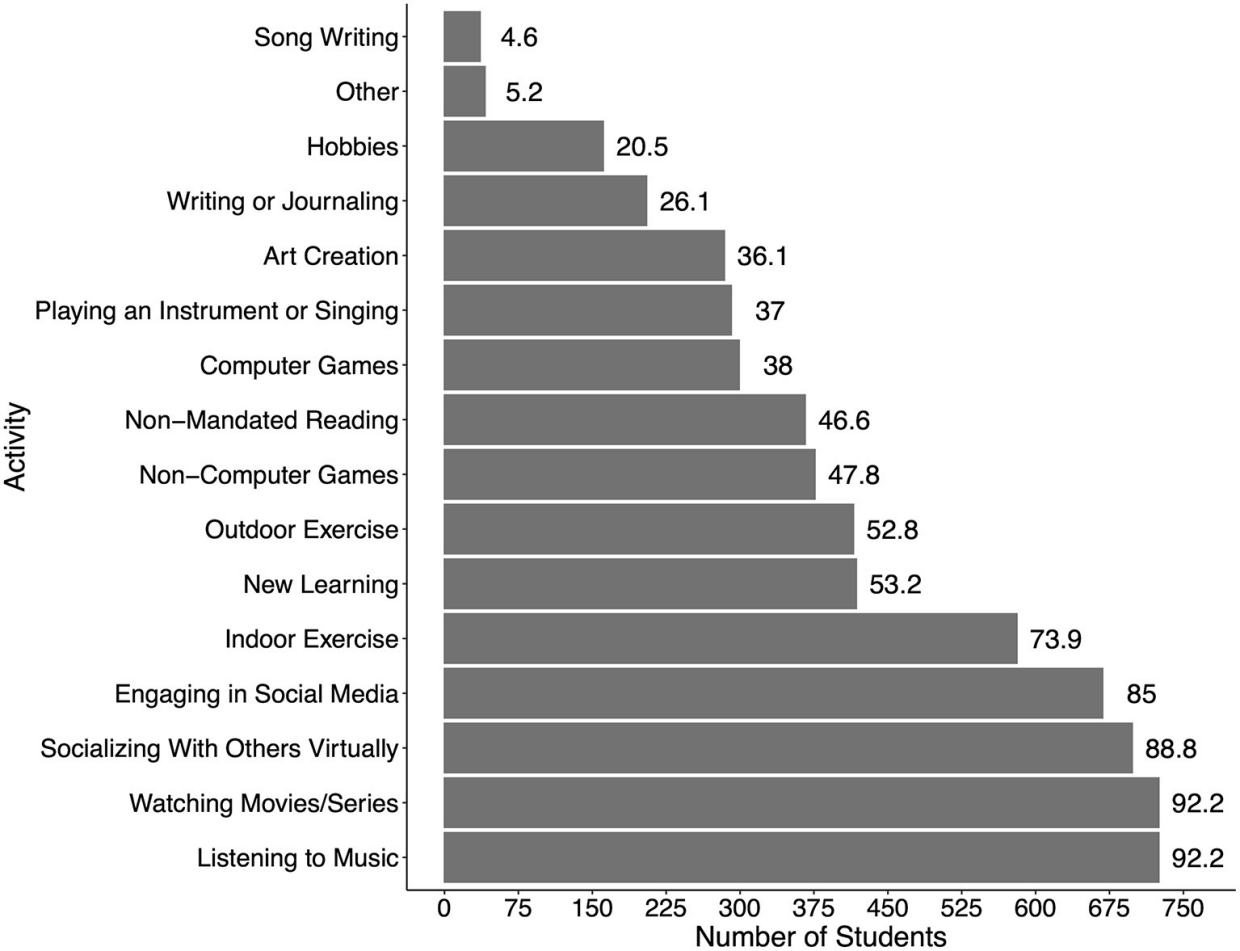
Number of students out of 786 who reported engaging in each extra-curricular activity. Numbers at the end of each bar indicate the % of students.

A survey was created (see Supplementary Materials for the full survey) consisting of 35 questions related to demographics, musical background, and participation in extra-curricular activities, followed by a question on interest in participating in group online music, art, and verbal therapy, an open-ended question on their experiences during the COVID-19 restrictions, and ending with two standardized questionnaires on anxiety levels (STAI-S) and personality traits (TIPI). Concerning extra-curricular activities, students first indicated whether they participated in each of the 16 extra-curricular activities listed (with the option to add additional activities not on the list). The list of activities can be seen in Figure 1. For each activity in which they participated, students were asked to give subjective ratings of how much they felt that each activity supported their overall well-being during the COVID-19 precautions (1 = not at all; 7 = extremely). For each activity students reported participating in, they were asked if they participated in the activity alone, with those in their household, or with others over the internet. Students were also asked if they participated in any activities more presently than prior to the COVID-19 precautionary measures. To gauge interest in proactive online group therapies, students were asked whether they would be interested (yes, no, maybe) in participating in each of group music, art, and verbal therapy. It was mandatory to complete each question in order to move through the survey (but sensitive questions included the response option “prefer not to answer”).

There were two optional questions. Students were asked whether they had sought mental health support and, if so, the type of support. Finally, in an open-ended question, students were asked to describe their experiences during the COVID-19 lockdown.

#### State-Trait Anxiety Inventory (STAI-S)

The State-Trait Anxiety Inventory (STAI) is a self-report questionnaire created to measure a person's level of anxiety. The original inventory (Form X) was revised in 1979 to replace items that had weak psychometric properties for certain groups, to better discriminate between feelings of anxiety and depression, and to improve its factor structure (Spielberger et al., 1983). The updated inventory (Form Y; Spielberger et al., 1980) was used in the present study. The STAI consists of two forms. The Trait form (STAI-T) measures individual differences in anxiety proneness and a person's general anxiety levels, while the State form (STAI-S) measures the intensity of the participant's anxiety at the moment of testing, in the recent past, or how they anticipate they would feel in a hypothetical situation (Spielberger et al., 1983). We asked students to answer the questions based on their present state. Each form consists of 20 statements. On the STAI-S, participants rate the intensity of their feelings on a Likert scale from (1) not at all to (4) very much so. Spielberger et al., collected normative adult data for these forms from 4 different groups: working adults (*N* = 1,838), college students (*N* = 855), high school students (*N* = 424), and military recruits (1964). The STAI-S showed good reliability and validity across the different normative groups; Cronbach's alpha = 0.86–0.95, and median item-remainder correlation = 0.55–0.63 (Spielberger et al., 1983). Construct validity was tested in two studies by comparing the mean STAI-S scores of college students in anxiety-inducing conditions (e.g., how they would feel just before an important exam, after being interrupted on the third administration of the Concept Mastery Test (Terman, 1956), or after watching a stressful movie) to mean STAI-S scores in control or relaxed conditions (e.g., normal form procedures, after a relaxation training period). In both studies, the anxiety-inducing conditions resulted in higher scores (Spielberger et al., 1983). The adequate reliability and validity of this instrument across different populations was also confirmed in a paper summarizing reported psychometric properties reported in 75 studies using the STAI (Barnes et al., 2002). In the present study, the STAI-S scores were used to examine students' anxiety levels, and as the dependent variable in exploratory regression analyses.

#### Ten-Item Personality Inventory

The Ten-Item Personality Inventory (TIPI) is a self-report questionnaire that measures a person's Big Five personality dimensions: extraversion, agreeableness, conscientiousness, emotional stability, and openness to experiences (Gosling et al., 2003). The questionnaire consists of 10 pairs of words (e.g., extraverted, enthusiastic). Participants rate the extent that each pair of traits applies to themselves on a Likert scale from (1) disagree strongly to (7) agree strongly. The TIPI has been shown to have good validity: mean convergent r with the Big-Five Inventory = 0.77, convergent r with the NEO Personality Inventory-Revised = 0.56–0.68, mean test-retest reliability = 0.72 (Gosling et al., 2003). Norms were also established with this sample (*N* = 1,813) (Gosling et al., 2003). In the present study, the TIPI was used to see if personality traits were related to anxiety levels and which extra-curricular activities students engaged in.

### Procedure

Students were informed that the survey would take ~20 min to complete. The initial page of the survey contained the letter of information and students gave consent online. The survey was anonymous, and students were provided the option to withdraw from the survey at any time while remaining eligible for the draw (see Participants section). After the submission of the survey (or if they chose not to complete) students were provided with links to mental health resources and the option to enter the draw. The survey was made available from April 21st−30th 2020, coinciding with the exam period at McMaster University.

### Data Analysis

First, descriptive statistics of students' responses were collated to answer our first three questions: Question 1: Which extra-curricular activities did undergraduate students engage in on their own volition during this time? Question 2: Which activities did students report as contributing to their wellness? Question 3: How anxious were students as measured by the standardized STAI-S during this time?

Second, exploratory analyses using linear regression models in R version 4.0.2 (R Core Team, 2019) were conducted to examine the following questions: Question 4: How did participating in each key activity relate to anxiety scores? Question 5: Did social aspects—participating in activities alone or with others—impact wellness and anxiety? Question 6: Did students' ratings of how key activities affected their well-being relate to their state anxiety scores? Question 7: Did students' personality traits relate to their state anxiety scores or the activities they chose to participate in? Each model was fitted using STAI-S scores as the dependent measure. To control for the fact that women report higher stress levels than men (Bayram and Bilgel, 2008; Al-Qaisy, 2011) we included participant gender as a control variable in each model. To ensure proper fit, we removed any students who either did not report their gender (four students) or reported a gender other than male or female (four students) as there was not enough data in these categories to analyze. This resulted in data from 778 students being included in the models. Furthermore, we included the result of the question “Have you ever been diagnosed with a neurological/psychological disorder?” as a control variable. We chose to focus on nine key activities out of the 16 in the survey based on two criteria: they were chosen by a substantial number of students and/or were related to a priori predictions concerning music and well-being. The nine key activities analyzed were (1) playing an instrument or singing, (2) listening to music, (3) writing songs, (4) participating in social media, (5) watching movies and TV series, (6) playing computer games, (7) outdoor exercise, (8) indoor exercise, and (9) journaling. For each question, single models were created for each of the nine key activities. These models are exploratory; we corrected for multiple comparisons using the Bonferroni correction.

Third, answers to the question on interest in participating in online group music, art, and verbal therapy were tabulated to answer Question 8: How much interest did students report in participating in online group music, art and verbal therapy?

Finally, to answer Question 9: How did students describe their experiences during COVID-19 precautionary measures, answers to the open-ended question inviting students to describe their experiences during the precautionary measures of the first wave of the COVID-19 pandemic were independently categorized by two researchers. Initial searches indicated that comments could be categorized at a high level into positive and negative categories. Each researcher further independently identified subthemes within each of the positive and negative categories. On amalgamation of their results, 3 positive subthemes and 3 negative subthemes were identified.

## Results

### Descriptive Analyses

#### Question 1: Which Extra-Curricular Activities Did Undergraduate Students Engage in of Their Own Volition During This Time?

**Figure 2:**
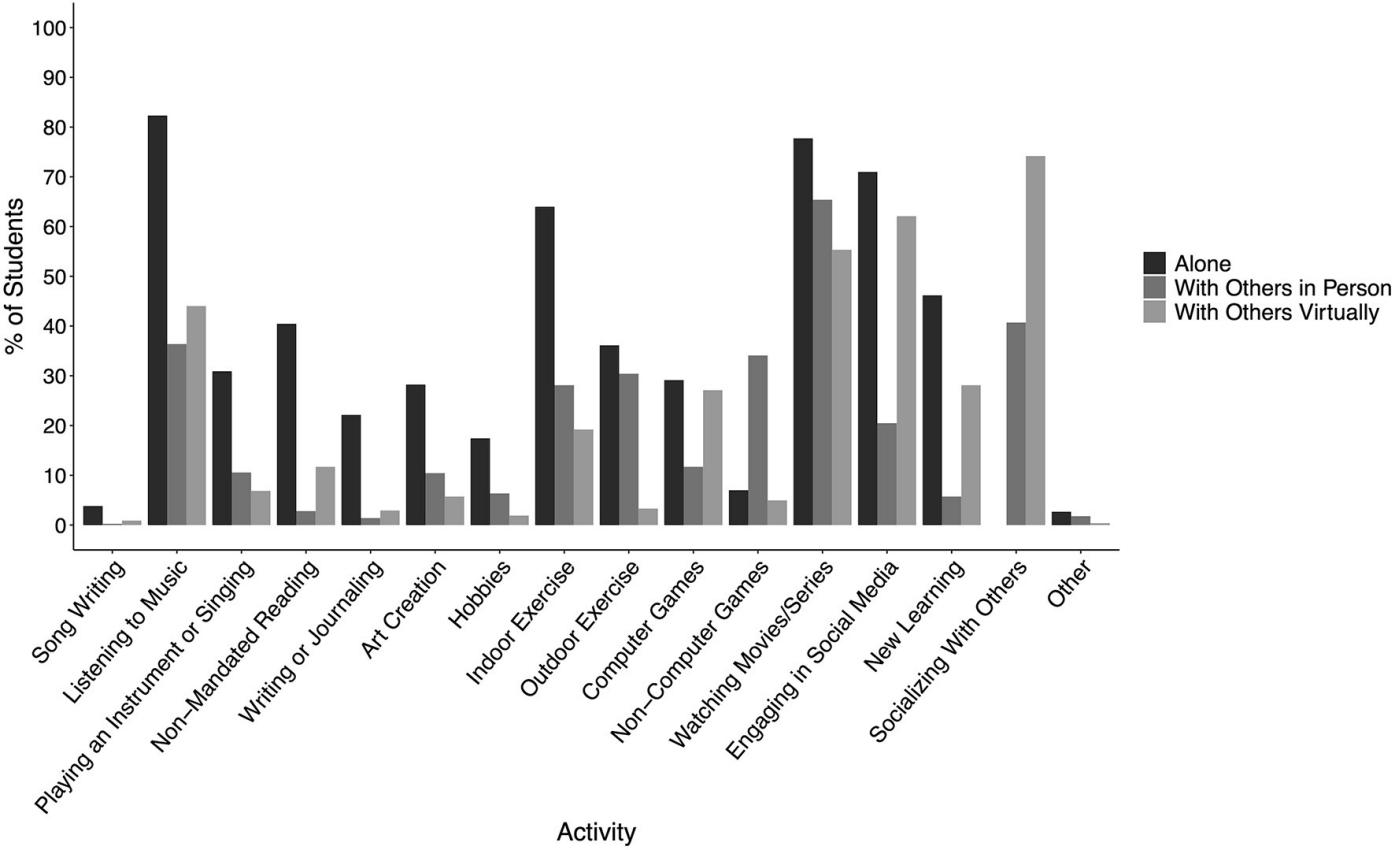
Percent of students out of 786 who reported engaging in each extra-curricular activity alone, with others in person or with others over the internet. Note that students could engage in a particular activity in more than one way.

The number of students who reported engaging in each of the 16 extra-curricular activities is shown in Figure 1. It can be seen that the two top activities were listening to music (92% of students) and watching movies/series (92%), followed by socializing with others over social media or phone (89%). Listening to music was the top activity that students engaged in alone (82%), socializing with others virtually using social media or phone was the top activity engaged in with others over the internet (74%), and watching movies was the top activity engaged in with those in the same living space (65%). A full list of how students engaged in each activity is presented in Figure 2.

#### Question 2: Which Activities Did Students Report as Contributing to Their Wellness?

**Figure 3:**
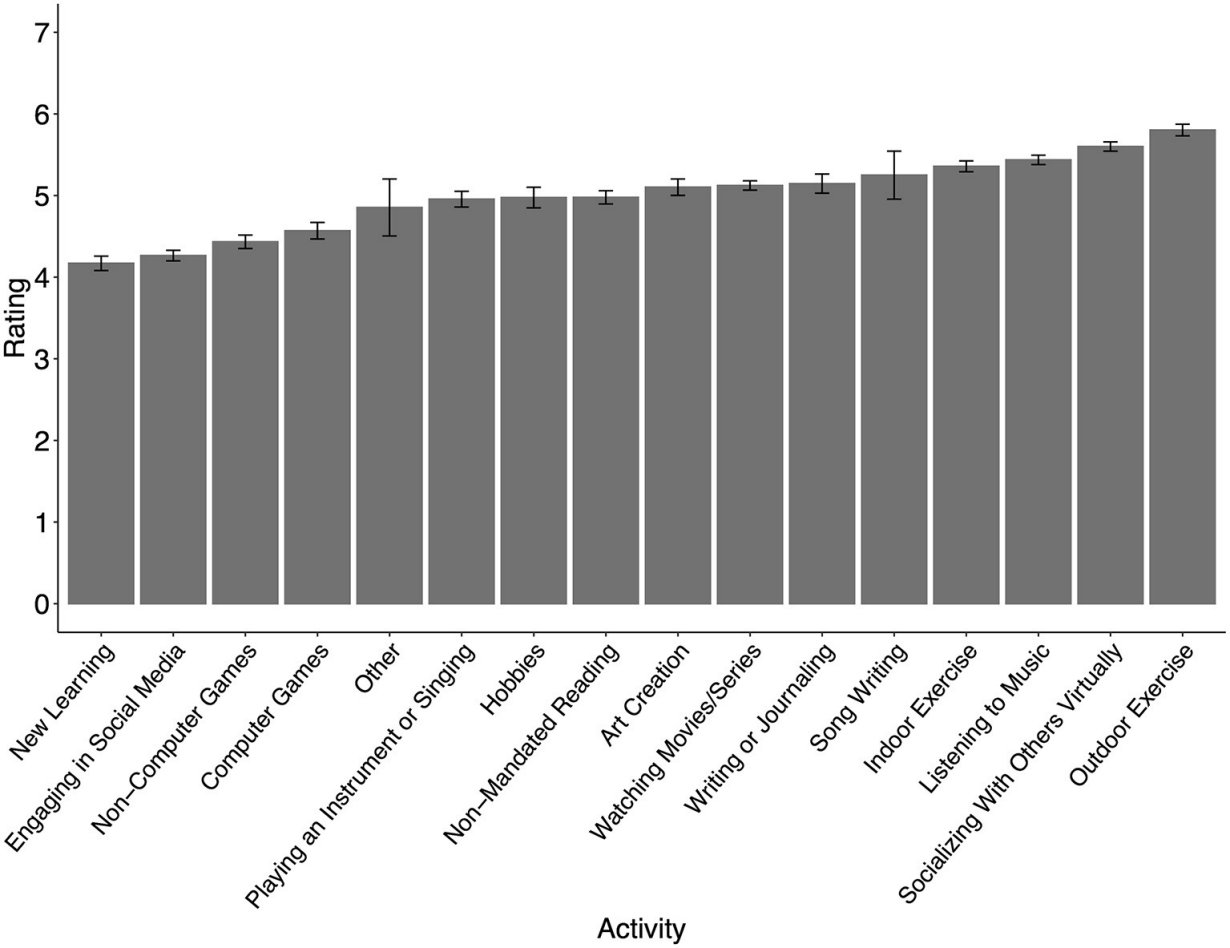
Mean ratings out of 7 for how helpful each extra-curricular activity was rated to be for well-being. Note that for each extra-curricular activity, only those students who reported engaging in the activity were included in the ratings for that activity. Error bars represent standard error.

For each extra-curricular activity that students reported engaging in, they rated how helpful that activity was for their well-being on a scale from 1 (not helpful) to 7 (extremely helpful). Mean ratings for each activity are shown in Figure 3. The top-rated activities were outdoor exercise (M = 5.8, SE = 0.07), socializing with others through social media or phone (M = 5.6, SE = 0.06), and listening to music (M = 5.4, SE = 0.06).

#### Question 3: How Anxious Were Students as Measured by the Standardized STAI-S During This Time?

**Figure 4:**
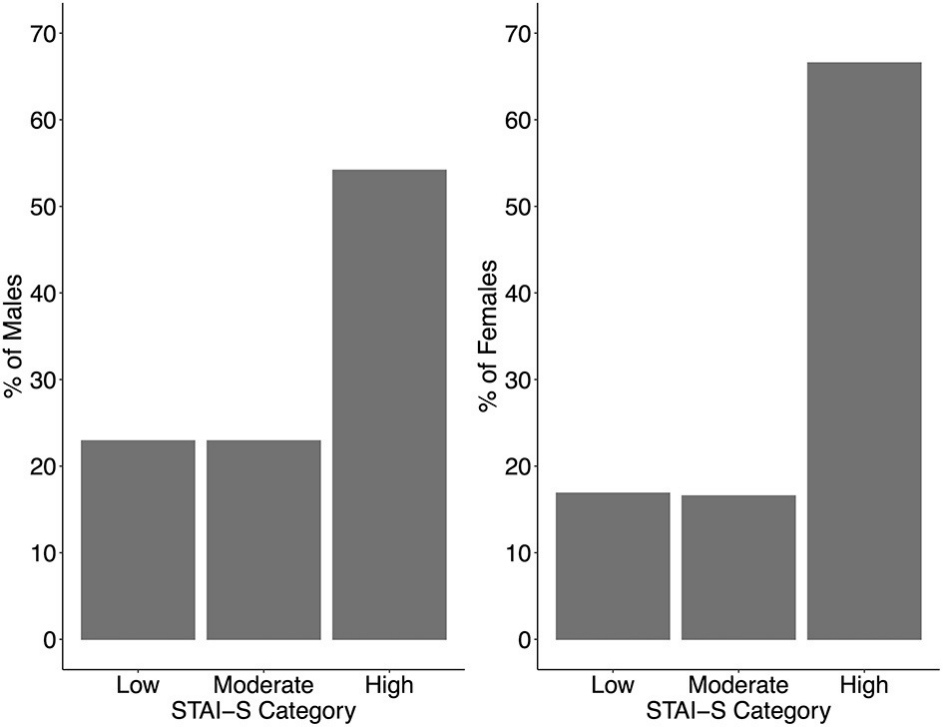
Percentage of males (*n* = 144; left panel) and females (*n* = 634; right panel) scoring in each category of the State Trait Anxiety Inventory—State subscale. Note: the students with gender other than male or female all scored in the High STAI-S category (*n* = 4).

Scores on the STAI-S revealed that, as a group, students showed high anxiety (Figure 4). 507 students (65%) scored in the high anxiety category. By gender, 67% of female students compared to 54% of male students scored in this high anxiety category. Females also had a higher mean STAI-S score than males [females: M = 50.89, SE = 0.50; males: M = 45.58, SE = 0.95; *t*_(227.63)_ = 4.67, *p* < 0.001]. 187 students (24%) reported they had reached out for supports through the McMaster University Wellness Centre or another professional support system. Together these scores suggest that students were highly stressed during the precautionary measures imposed during the first wave of the pandemic.

### Exploratory Regression Analyses

#### Question 4: How Did Participating in Each Key Activity Relate to Anxiety Scores?

With this group of models, we were interested in the effect of participating in each activity on a participant's state anxiety score. We dummy-coded whether the participant reported participating in the activity or not during the initial wave of the COVID-19 pandemic. As noted in the statistical analyses section in the Methods, participant gender and whether they reported having a psychological disorder were included as control variables. No activity significantly predicted a change in state anxiety after controlling for multiple comparisons (see Table 1 for the main effect of each activity). There was a significant effect of gender and whether they reported having a psychological disorder on their STAI-S score in every model. Watching movies/series during the start of the pandemic was associated with a lower STAI-S score by 3.6 points (*t* = −2.22; uncorrected *p* = 0.027; corrected *p* = 0.24). In this model, men reported a state anxiety score of 4.48 points lower than women (*t* = −3.99; uncorrected *p* < 0.001; corrected *p* < 0.001). Furthermore, students who reported a psychological disorder had an anxiety score that was 6.11 points higher than students without a psychological disorder (*t* = −3.99; uncorrected *p* < 0.001; corrected *p* < 0.001). Thus, future research could look at the effect of watching movies and TV series on anxiety during times of high stress.

**Table 1:** Effect of participating in each key activity on state anxiety scores (corrected *p*-values use Bonferroni correction for the 9 models examined).

**Variable**	**Estimate**	**Std. error**	***T*-value**	***P*-value**	**Corrected *p*-value**
Instrument playing and singing	0.45	0.91	0.05	0.96	N/A
Listening to music	−1.93	1.65	−1.17	0.24	N/A
Song writing	1.60	2.22	0.72	0.47	N/A
Social media	0.83	1.24	0.67	0.50	N/A
Movies/Series	−3.60	1.62	−2.22	0.027	0.24
Computer games	−1.75	0.95	−1.84	0.067	0.60
Outdoor exercise	−0.32	0.88	−0.36	0.72	N/A
Indoor exercise	−1.65	1.00	−1.65	0.099	0.891
Journaling	0.25	1.02	0.25	0.80	N/A

#### Question 5: Did Social Aspects—Participating in Activities Alone or With Others—Impact Anxiety?

With this group of models, we were interested in whether there was a benefit to participating in the extra-curricular activities with other individuals. We asked students whether they did each activity alone, with others in person, or with others online. We coded individuals as “participating with others” if they responded yes to either “with those in self-isolation with you” or “socially using the internet,” even if they reported participating in the activity alone. A negative estimate indicates that performing an activity with others (compared to doing so alone) is associated with lower STAI-S scores. As noted in the statistical analyses section in the Methods, participant gender and whether they reported having a psychological disorder were included as control variables. These two variables were consistently predictive of STAI-S score. After correcting for multiple comparisons, there were no significant differences in any of the activities (see Table 2 for the main effect of each activity). Playing computer games with others resulted in a STAI-S score of 4.97 points less than those who played alone (*t* = −2.67; uncorrected *p* = 0.008; corrected *p* = 0.072). Engaging in indoor exercise with others was associated with a STAI-S score of 2.84 points lower than doing indoor exercise alone (*t* = −2.66; uncorrected *p* = 0.008; corrected *p* = 0.072). Future research can investigate the effects of playing computer games and exercising indoors with others on state anxiety during a time of crisis.

**Table 2:** Effects of participating alone or with others on state anxiety scores (with alone as the reference point; corrected *p*–values use Bonferroni correction for the 9 models examined).

**Variable**	**Estimate**	**Std. error**	***T*-value**	***P*-value**	**Bonferroni corrected *p*-value**
Instrument playing and singing	−2.97	1.55	−1.92	0.056	0.50
Listening to music	0.11	1.04	0.11	0.92	N/A
Song writing	−1.41	5.72	−0.25	0.81	N/A
Social media	−1.76	1.21	−1.45	0.15	N/A
Movies and TV series	02.17	1.50	−1.45	0.15	N/A
Computer games	−4.97	1.86	−2.67	0.0080	0.072
Outdoor exercise	0.31	1.35	0.23	0.82	N/A
Indoor EXERCISE	−2.84	1.07	−2.66	0.0080	0.072
Journaling	0.67	2.81	0.24	0.81	N/A

#### Question 6: Did Students' Ratings of How Key Activities Affected Their Well-Being Relate to Their State Anxiety Scores?

With this group of models, we were interested in the relation between state anxiety scores and students' perception of an activity's helpfulness on their well-being. In the questionnaire, students rated how well each activity supported their overall well-being during the COVID-19 precautions on a scale from 1 (not at all) to 7 (extremely). A negative estimate indicates that with increases in how helpful a particular activity was rated for well-being, STAI-S scores decreased. As noted in the statistical analyses section in the Methods, participant gender and whether they reported having a psychological disorder were included as control variables. For three activities (social media, outdoor and indoor exercise), student reports that the activity was helpful for well-being were associated with lower STAI-S scores (see Table 3 for the main effect of each activity). For every 1-point increase in well-being rating for social media, STAI-S scores were lower by 0.90 points (*t* = −3.72; uncorrected *p* < 0.001; corrected *p* < 0.01). For every 1-point increase in well-being rating for outdoor exercise, STAI-S scores were lower by 1.4 points (*t* = −3.39; uncorrected *p* < 0.001; corrected *p* < 0.01). For every 1-point increase in well-being rating for indoor exercise, STAI-S scores were lower by 1.41 points (*t* = −4.44; uncorrected *p* < 0.001; corrected *p* < 0.001). Thus, finding these activities helpful for well-being was associated with lower anxiety. In addition, for all activities, the direction of the effect was in the appropriate direction, indicating that higher helpfulness ratings on each activity were associated with lower anxiety scores, although most did not reach significance.

**Table 3:** The effect of students' ratings of how effective key activities were on their well-being as predictors of STAI–S scores (corrected *p*-values use Bonferroni correction for the 9 models examined).

**Variable**	**Estimate**	**Std. error**	***T*-value**	***P*-value**	**Corrected *p*-value**
Instrument playing and singing	−0.72	0.44	−1.62	0.11	0.99
Listening to music	−0.32	0.30	−1.07	0.28	N/A
Song writing	−0.33	1.38	−0.24	0.81	N/A
Social media	−0.90	0.24	−3.72	0.00021	0.0019
Movies and TV series	−0.26	0.30	−0.86	0.39	N/A
Computer games	−0.57	0.42	−1.37	0.17	N/A
Outdoor exercise	−1.40	0.41	−3.39	0.00078	0.0070
Indoor exercise	−1.41	0.32	−4.44	0.000011	0.0001
Journaling	−0.55	0.56	−0.97	0.33	N/A

#### Question 7: Did Students' Personality Traits Relate to Their State Anxiety Scores or the Activities They Chose to Participate in?

With this group of models, we were interested in the relation between each of the five personality traits (as measured by the TIPI on a scale from 1 to 7) on students' state anxiety scores and on particular extra-curricular activities they chose to engage in. As noted in the statistical analysis section in the Methods, participant gender and whether they reported having a psychological disorder were included as control variables. We included all five TIPI traits as main effects. Beta-values for each TIPI trait represent the change in STAI-S score with a one-point increase on the 7-point personality trait scale. Individuals who scored high in conscientiousness or emotional stability had lower STAI-S scores (conscientiousness: *B* = −0.900, *t* = −2.89, *p* = 0.0039; emotional stability: *B* = −4.85, *t* = −17.19, *p* < 0.001).

We built additional binomial generalized linear models to look at the effect of TIPI traits on participation in the various extra-curricular activities during the COVID-19 lockdown. We included participants' gender and the presence/absence of a psychological disorder as control variables, and included all five TIPI variables as our main effects of interest. *P*-values were corrected to account for multiple comparisons (running models on the nine activities of interest). Individuals who were higher in openness to experience were more likely to participate in song writing (*B* = 0.453, *z* = 2.27, uncorrected *p* = 0.023; corrected *p* = 0.21), journaling (*B* = 0.315, *z* = 3.56, uncorrected *p* = 0.00037; corrected *p* = 0.0033) and playing an instrument or singing (*B* = 0.347, *z* = 4.38, uncorrected *p* < 0.001; corrected *p* < 0.001). Individuals who were higher in conscientiousness were more likely to participate in indoor exercise (*B* = 0.260, *z* = 3.60, uncorrected *p* = 0.00032; corrected *p* = 0.0029) and social media (*B* = 0.179, *z* = 2.05, uncorrected *p* = 0.040; corrected *p* = 0.36), and were less likely to play video or computer games (*B* = 0.224, *z* = *B*=3.24, uncorrected *p* = 0.0012; corrected *p* = 0.011). Individuals who were higher in extraversion were more likely to participate in indoor exercise (*B* = 0.156, *z* = 2.63, uncorrected *p* = 0.0086; corrected *p* = 0.078), outdoor exercise (*B* = 0.114, *z* = 2.22, uncorrected *p* = 0.0262; corrected *p* = 0.24), and social media (*B* = 0.159, *z* = 2.16, uncorrected *p* = 0.031; corrected *p* = 0.28). Individuals who were higher in emotional stability were more likely to participate in outdoor exercise (*B* = 0.120, *z* = 2.03, uncorrected *p* = 0.042; corrected *p* = 0.38) and individuals who were higher in agreeableness were less likely to play video or computer games (*B* = 0.161, *z* = 2.14, uncorrected *p* = 0.033; corrected *p* = 0.29). No personality traits predicted watching movies or TV series or listening to music. Together these analyses indicate that there are large individual differences related to personality traits in students' choices of extra-curricular activities, suggesting that to engage the maximum number of students in proactive wellness initiatives, a variety of programs would be beneficial.

### Interest in Online Group Music, Art, and Verbal Therapy

#### Question 8: How Much Interest Did Students Report in Participating in Online Group Music, Art, and Verbal Therapy?

**Figure 5:**
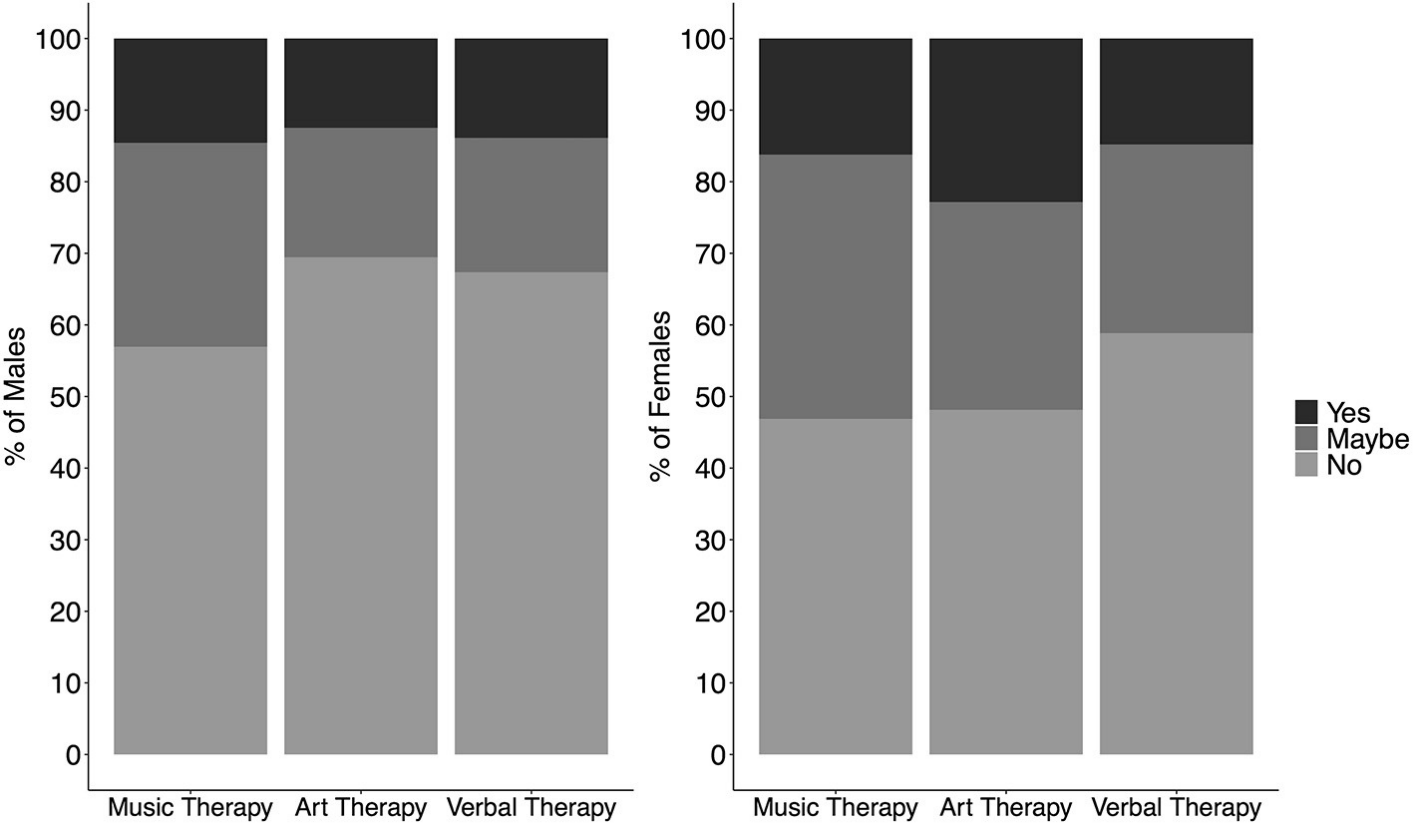
Percentage of males (*n* = 144; left panel) and females (*n* = 634; right panel) indicating if they were interested, maybe interested, or not interested in three types of drop-in online therapy: music, art, and verbal. Note that out of students with gender other than male or female (*n* = 4), 1 said No to Music Therapy, 3 said Yes to Music Therapy, 1 said No to Art Therapy, 3 said Yes to Art Therapy, 3 said No to Verbal Therapy, and 1 said Yes to Verbal Therapy).

When asked about interest in participating in three types of online group therapy, 52% indicated that either they were interested or were maybe interested in participating in music therapy. The percentage for art therapy was comparable at 48%. The percentage for verbal therapy was lower at 40%. The complete breakdown by gender can be seen in Figure 5. Together these results suggest considerable interest in group music and art therapy among university students.

### Content Analysis of Open-Ended Responses

#### Question 9: How Did Students Describe Their Experiences During COVID-19 Precautionary Measures in an Open-Ended Format?

145 students (18%) responded to the open-ended question “*Please add any additional information that you feel comfortable sharing about how you are coping at this time of change as a result of the COVID-19 pandemic.”* Comments were fairly evenly split between expressing positive (73 comments) and negative (78 comments) experiences (see Table 4). A few students wrote more than one comment, giving rise to a total of 151 comments analyzed. Within the positive category, three subthemes surfaced: (1) positive impact of engaging in self-care (*n* = 15 comments), (2) positive impact of engaging in activities (*n* = 40), and (3) impact of keeping a positive attitude (*n* = 18). Three subthemes also surfaced within the negative category: (1) negative impact on studies (*n* = 24), (2) negative impact on well-being (*n* = 45), and (3) negative impact due to living arrangements (*n* = 9). The full set of responses in each subtheme is available in Supplementary Materials, Section B. Table 4 shows an example response for each subtheme.

**Table 4:** Example responses to the open-ended question falling into each of the three positive and three negative subthemes.

**Subtheme**	**Example response**
Positive impact on self-care	I am just trying to take care of myself. I enjoy listening to music and socializing with my family members, who I live at home with. It is such a tough time and trying to learn something new is what I have been doing—whether if it is a cool little fact or something new about myself as a person
Positive impact of engaging in activities	I started a couple of personal projects such as a video series and a story
Positive impact on attitude	Each day is a bit different, some are better and some are worse but I'm trying to stay optimistic. One really great thing about being home is that I can spend more time with family and on hobbies, but on the other hand, I really miss my friends and boyfriend.
Negative impact on studies	Exams have been insanely anxiety provoking and stressful after having been isolated and learning online.
Negative impact on well-being	I find it very hard to be motivated to do essentially anything. I stay in bed until 1 every day because I know that the day will be boring when I eventually get up.
Negative impact due to living arrangements	Difficult to be at home due to family issues, hard to be away from friends and other loved ones

## Discussion

It is recognized that participating in extra-curricular activities have cognitive, social and mental health benefits (Fares et al., 2016), and this may be even more important during stressful times when social interactions are limited. In our survey, the most popular extra-curricular activities students reported engaging in during the pandemic lockdown were listening to music (92% of students), watching movies/series (92%), socializing with others virtually (89%), and engaging in social media (85%) (see Figure 1). Interestingly, these activities were engaged in by a greater number of students than indoor exercise (74%) or outdoor exercise (53%), despite exercise being an activity that is known to lower stress (Reed and Buck, 2009; Rebar et al., 2015). One limitation in interpreting these numbers, however, is that we did not gather data on how often or for what duration of time students engaged in these activities. Nor do we know how the types of activities engaged in may have changed pre to post pandemic, or whether the amount of time spent engaging in each activity changed compared to pre-pandemic levels. Nonetheless, it is interesting that listening to music was one of the two activities engaged in by the most students during this time, suggesting that music was important to them.

Also of interest was that watching movies/series, socializing with others virtually, and engaging in social media were done to a large extent with others, either in person or virtually (see Figure 2), compared to alone. Even for music listening, 36% of students reported engaging in this activity with others in person and 44% reported engaging in this activity with others virtually (see Figure 2). Together these numbers suggest that the activities students were engaging in the most tended to be ones in which they incorporated a social component. In turn, this suggests that extra-curricular activities were serving the important function of increasing students' social interactions with others.

When asked how much students felt that each extra-curricular activity contributed to their well-being, the mean ratings for all of the 16 activities were above 4 on a scale from 1 (not helpful) to 7 (extremely helpful), and the mean across all activities was 5.0. This corroborates previous literature on the positive impact of extra-curricular activities on wellness (Fares et al., 2016). The data from the present study suggests, further, that this is also true during a highly stressful time involving social isolation. Despite the fact that only 53% of students reported engaging in outdoor exercise, it received the highest rating (5.8 ± 0.07) for contributing to well-being. This is consistent with many studies showing that engaging in exercise is beneficial for mental health and well-being (Reed and Buck, 2009; Rebar et al., 2015). Interestingly, exercising indoors (5.4 ± 0.07) was rated as having lower benefit than exercising outdoors, despite the fact that more students engaged in indoor exercise. One speculation is that this relates to the fact that students engaged in outdoor exercise to a greater extent with others than was the case with indoor exercise, again suggesting that during the pandemic, activities incorporating social interaction were felt by students to lead to the most benefit. Consistent with this is also that ratings were high for the perceived well-being benefits of socializing with others virtually (5.6 ± 0.06) and listening to music (5.4 ± 0.06), both of which were engaged in to large extent with others, whether virtually or in person (see Figure 2).

To obtain a more objective indication of students' anxiety during the convergence of the initial pandemic lockdown and a newly virtual exam period, students completed the STAI-S. Scores were alarmingly high, with 65% scoring in the high anxiety category (see Figure 4). The expected norms for state anxiety by gender are 36.47 for males and 38.76 for females (Spielberger et al., 1983). The cut off for the high anxiety category is commonly classified as 45 and above (Kayikcioglu et al., 2017) and the students in the present study had an average of 49.69 with more females (67%, *M* = 50.59) than males (54%, *M* = 45.58) falling in the high anxiety category, consistent with previous findings (Moyser, 2020). However, interpretation of these results needs to be qualified by several factors. First, it is possible that the sample was biased. For example, students with mental health concerns might have been more likely to complete the survey; alternatively, students with mental health concerns may have avoided completing the survey. Second, many more female than male students completed the survey (82%), despite the percentage of female students at McMaster being 63%. It is possible that there is a greater stigma for male students than for female students to think about or admit to challenges related to their well-being (Chatmon, 2020). It is also possible that the lower STAI-S scores in males in the present study reflects that those with concerns about their mental health chose not to participate. Interestingly, the number of students who reported reaching out for support through the McMaster University Wellness Centre or other professional support systems was only 24%, despite 67% scoring in the high anxiety category of the STAI-S. This suggests that many students who may have benefitted from a mental health intervention were not getting professional support during this time.

A series of exploratory analyses revealed that STAI-S scores were not significantly related to participation in any particular key extra-curricular activity after controlling for multiple comparisons. However, in general, both reporting to have a psychological disorder and being female were associated with higher STAI-S scores. This suggests that the STAI-S scores may have reflected individual differences in trait anxiety to a greater extent than whether particular extra-curricular activities lowered anxiety during the stressful pandemic circumstances. We were of course unable to obtain STAI-S scores pre-pandemic, so it was impossible to examine how anxiety scores *changed* from before to during the pandemic in relation to what activities students chose to engage in. As well, the study was exploratory in that we did not have random assignment to participate in particular extracurricular activities. It thus remains for future studies to examine how various extra-curricular activities may moderate changes in anxiety when students are placed in stressful circumstances.

Similarly, exploratory analyses concerning whether participating in particular key activities alone or with others (whether in person or virtually) found no effects after controlling for multiple comparisons. However, there were trends that playing computer games with others and that engaging in indoor exercise with others were associated with lower STAI-S scores when compared to participating in these activities alone. The same caveat applies here of course, that we do not have pre-pandemic STAI-S scores, so we cannot determine whether participating in particular activities socially during the lockdown was associated with a smaller pre to during *change* in anxiety compared to participating in those activities alone.

Exploratory analyses of whether STAI-S scores related to students' ratings of how participation in key activities affected their well-being revealed a positive relationship for every activity, but only three of these reached significance levels after correction for multiple comparisons: engaging in social media, indoor exercise and outdoor exercise. Again, if we had been able to obtain STAI-S scores before and during the lockdown, and been able to examine changes in anxiety, it is possible that stronger relations might be found between students' ratings of how effective an activity was and how much their anxiety changed pre to during pandemic lockdown.

These exploratory analyses all suggest that individual differences likely play a large role in how students respond to stress, which activities they find helpful for their well-being, and the extent to which social aspects affect their well-being. As a preliminary foray into examining this question, we investigated how the five personality traits measured by the TIPI related to STAI-S anxiety scores, as well as to the activities participants chose to engage in. We first noted that students who scored high in conscientiousness or emotional stability had lower STAI-S scores. We are unable of course to determine whether this represents lower trait anxiety or a superior ability to cope with stress. However, the findings are consistent with Bunevicius et al. (2008) that emotional stability is related to anxiety and with Strickhouser et al. (2017), that conscientiousness is related to anxiety. Of interest here is that participation in some particular extracurricular activities was associated with particular personality traits. In particular, participating in exercise was significantly higher in individuals scoring high in conscientiousness, extraversion or emotional stability. Engaging in journaling and playing an instrument or singing were significantly higher in individuals who scored high on openness to experience, with a trend for higher engagement in song writing. No personality traits were associated with listening to music or to watching movies or TV. However, the vast majority of students engaged in these activities, so a lack of association with personality traits may reflect ceiling effects. In future studies, measures of how often students engage in these activities, rather than simply whether they engage in them or not, might be more sensitive at picking up how they relate to personality differences. What is clear from the present data is that listening to music and watching movies or TV are very common extracurricular activities regardless of personality. In general, from the present study, we cannot determine whether particular extracurricular activity choices were similar pre to during the pandemic, but the important point is that individual differences likely lead people to participate in different extracurricular activities, all of which may be beneficial for their mental health.

Further to the question of individual differences, we found a variety of responses when students were simply asked to share their experiences during the COVID-19 precautionary measures. We were surprised to find that about half the responses were positive while half were negative. For example, some students found living at home a positive experience while others found it a negative experience. Some students were happy to have time to engage in particular activities, whether cooking or engaging in self-care, whereas others keenly felt the negative impact of in-person isolation from their friends.

The question of individual differences is critical when thinking about providing mental health supports for students on university campuses. University students are at a vulnerable age for mental health struggles compared to the general population (Lee and Jung, 2018), a situation likely exacerbated by the pandemic lockdown. Currently, university mental health supports in Canada tend to be limited to verbal-based therapies. According to the Canadian Alliance of Student Associations, wait times to see a campus counselor can be up to 2–3 months, potentially contributing further to a mental health crises that might have otherwise been prevented (Max and Waters, 2018). In 2016, 40% of individuals under the age of 24 who visited an emergency department for a mental illness or addiction problem in Ontario had not received mental health care from a family doctor, pediatrician, or psychiatrist within the past 2 years (Health Quality Ontario, 2018). Three considerations arise from this situation. First, there needs to be a way to *scale up* mental health supports to reach more students. Second, a *proactive* approach is needed, so that students have supports for managing stress and anxiety before crises are reached. And, third, *individual differences* need to be considered, so that optimal supports for individual students are available.

The present survey was conducted in part to gauge students' needs and interest in additional alternative mental health supports on university campuses. In particular, we were interested in music- or art-based group therapies. Currently, such supports are rare on university campus, but such therapies could go some way to addressing the three considerations outlined here. Group therapies are cost effective and scale up easily, they can be delivered to students proactively prior to or without a specific diagnosis, and they could provide an alternative for students who prefer not to engage in individual therapy. In our survey, 52% of students indicated they either were interested or might be interested in group music therapy. A comparable percentage indicated interest in group art therapy (48%), while the percentage interested in verbal group therapy was lower at 40%. Combined with other findings from our survey—that students felt that engaging in musical extracurricular activities was beneficial for their well-being, that they often engaged in music listening with others (whether virtual or in person), that students tended to rate activities as more beneficial when they involved a social component, and that different personality traits were associated with which extracurricular activities students chose to engage in—this suggests that offering proactive group music therapy on university campuses would be of interest to a substantial number of students, and could be a cost effective way to provide a social, proactive mental health support to a significant subset of students for whom such an approach might be more optimal than verbal therapy. To this end, we are currently conducting a randomized control study using questionnaire and physiological measures to compare the benefits of participating in different types of group music therapies and group verbal therapy.

## Conclusions

A survey of over 700 students at a Canadian university during the pandemic lockdown in April 2020 revealed that students were feeling highly anxious, with 65% scoring in the high anxiety category of the standardized STAI-S measure. Previous studies have shown that participation in extracurricular activities is associated with cognitive, social and well-being benefits. Results of the present survey revealed that students engaged in a variety of extracurricular activities during this time, the most popular being listening to music, watching movies and series, socializing with others virtually, and engaging in social media. All extracurricular activities were rated as being beneficial for well-being, with the highest ratings going to outdoor exercise, socializing virtually with others and listening to music. Social interaction was important in these activities, with students reporting engaging with others (virtually or in person) in the activities reported to be most beneficial. After correction for multiple comparisons, there were few significant associations between STAI-S scores and self-report measures, likely because we did not have a pre-pandemic measure of STAI-S and therefore could not examine changes in STAI-S scores. However, it was clear that individual differences in the choice of, and perceived benefit of, different extracurricular activities were large. Individual differences in activity choice were significantly related to personality characteristics, with individuals higher in *conscientiousness* or *extraversion* or *emotional stability* more likely to engage in exercise, and those higher in *openness to experience* more likely to engage in journaling and playing a musical instrument or singing with a trend for higher engagement in song writing. That the current practice on university campuses of providing almost exclusively verbal therapy, and few options of proactive (i.e., before crisis) group therapies, is not meeting the needs of many students was evident in that 52% of respondents were either interested or maybe interested in proactive group music therapy and 48% in proactive group art therapy, while 40% were potentially interested in group verbal therapy. The results of this survey suggest that providing a variety of proactive social (group) therapy options for students would likely contribute substantially to students' mental health and well-being.

## Data Availability Statement

The raw data supporting the conclusions of this article will be made available by the authors, without undue reservation.

## Ethics Statement

The studies involving human participants were reviewed and approved by McMaster University Research Ethics Board. The patients/participants provided their written informed consent to participate in this study.

## Author Contributions

RF, SM, and LT conceived the research ideas and designed the survey. RF, SM, and CI analyzed the data. All authors contributed to writing the paper.

## Conflict of Interest

The authors declare that the research was conducted in the absence of any commercial or financial relationships that could be construed as a potential conflict of interest.
