# Supplementary material for: Extra-Curricular Activities and Well-Being: Results From a Survey of Undergraduate University Students During COVID-19 Lockdown Restrictions
Source: Front Psychol. 2021 Jun 28;12:647402. doi: 10.3389/fpsyg.2021.647402 (PMC8274476; doi:10.3389/fpsyg.2021.647402)
Supplement: Supplementary file 1 [file Data_Sheet_1.PDF]

Supplementary Materials

A. <u>The complete survey given to students</u>

EXTRA-CURRICULAR ACTIVITIES AND WELLNESS UNDER COVID-19 PRECAUTIONARY MEASURES

Purpose of the Study:
To gain insight into undergraduate students' engagement in extra-curricular activities and their wellbeing under COVID-19 precautionary measures.

Description of the research:
The COVID-19 precautionary measures implemented end of day Friday March 13th resulted in student classes transitioning to on-line platforms, cancellations of events and activities, social distancing and, for many, relocation of accommodations. The data collected in this questionnaire will inform how McMaster University can better support its students. You will be asked to complete a number of questions that includes demographic and health questions, questions about your musical background and your current extra-curricular activities. It should take approximately 30 minutes to complete.

Potential Risks:
There are no known harms associated with these procedures. You may choose to not answer questions on the questionnaires if they make you feel uncomfortable. There is a risk that someone else in the vicinity of your workspace or who has access to your computer could see your responses; to minimize this risk, it is recommended that you complete the questionnaire in a private place and clear your web browser's history after submitting your responses.

Potential Benefits:
Completing the questionnaire will not benefit you directly, but the intent is that the study will contribute to the development of meaningful supports for undergraduate students. If you are enrolled in an appropriate course, you will be able to obtain course credit as outlined in your course outline. After completing the study, you will have a choice to participate in a draw for one of ten $50 prizes. Odds of winning the draw will depend on how many people take part in the study. We expect about 500 participants in which case the odds of winning should be about 1 in 50. Only winners will be contacted and we expect this to happen before the end of May, 31 2020.

Confidentiality:
Confidentiality will be respected and no information that discloses your identity will be released or published without your consent, unless required by law (for example, laws pertaining to the protection of vulnerable individuals including children). Data collected by LimeSurvey is stored on secure servers at McMaster University. All data will remain in Dr. Trainor's lab, stored in de-identified form for possible future analysis, until such time as they are destroyed.

Participation:
Participation in research is voluntary. If you choose to participate, you can withdraw from the study at any time without prejudice, even after signing this form. In the case of withdrawal, all data will be destroyed unless you indicate otherwise.

Ethics Review:

This research has been approved by the McMaster Research Ethics Board (MREB). Participants with concerns or questions may contact the Ethics Secretariat, Research Office for Administration, Development and Support (ROADS), (905) 525-9140 ext. 23142, ethicsoffice@mcmaster.ca

Consent to Participate

I acknowledge that the research procedures have been explained to me and that any questions that I have asked have been answered to my satisfaction. I have been informed of the alternatives to participation in this study, including the right not to participate and the right to withdraw at any time without prejudice. As well, the potential harms and benefits of participating have been explained to me. I know that I may ask now, or in the future, any questions I have about the study or the research procedures. I have been assured that individual results will be kept confidential and that no information will be released or printed that would disclose my personal identity without my permission, unless required by law. Having read the above, I understand that by clicking the "Next" button below, I agree to take part in this study under the terms and conditions outlined in the accompanying letter of information.

- Next

There are 35 questions in this survey.

General Information

Please indicate your age.

- 18 or younger
- 19-20
- 21-22
- 23-24
- 25 or older

What is your gender:

- Male
- Female
- Prefer not to answer
- Prefer to self-describe
  Please enter your comment here:

Are you a full-time student?

- Yes
- No

Are you an international student?

- Yes
- No

How many hours per week are you currently spending on your coursework?

- 1-5 hours
- 5-10 hours
- 10-15 hours
- More than 15 hours

If you are employed, how many hours per week do you currently work?

- 1-5 hours
- 5-10 hours
- 10-15 hours
- More than 15 hours

Under which faculty are you studying?
- Science
- Humanities
- Social Science
- Business
- Engineering
- Health Sciences

What are your current accommodations?
- Detached house
- Semi-detached house
- Apartment/Condominium
- One room rental in shared accommodation
- Other. Please describe
  Please enter your comment here:

Please indicate with whom you are sharing your accommodations:
- Parent(s)
- Sibling(s)
- Friend(s)
- Partner
- Child(ren)
- I am living alone
- Other
  Please enter your comment here:

Have you ever been diagnosed with a neurological/psychological disorder?
- Yes
- No
- Prefer not to answer

Are you a McMaster University student?
- Yes
- If no, which university do you attend?
  Please enter your comment here:

Which year of study will you complete in April 2020?
- Year 1
- Year 2
- Year 3
- Year 4
- If other, please describe.
  Please enter your comment here:

Have you always lived in Canada?
- Yes

- No
- If not, where else have you lived?
  Please enter your comment here:

Can you play any musical instruments? If yes, please indicate ~~with~~ which instruments you play (including singing) and the level of training achieved on each.

Comment here:	Instrument 1	Level of training
	Instrument 2	Level of training
	Instrument 3	Level of training

Extra-Curricular Activities

Which of the following activities have you engaged in since the COVID-19 precautionary measures were implemented March 16, 2020? Please select all that apply.

- Song writing
- Listening to music
- Non-mandated reading (includes audiobooks & podcasts)
- Writing or journaling
- Art creation
- Hobbies such as knitting or model building
- Indoor Exercise
- Outdoor Exercise
- Computer gaming
- Non-computer games (board games or cards)
- Watching movies/series
- Engaging in social media
- New learning (online courses, webinars etc.)
- Socializing with others (through social media or phone)
- Other (please specify)
  Please enter your comment here:

Which of these activities have you engaged in alone? Please select all that apply.

- Song writing
- Listening to music
- Non-mandated reading (includes audiobooks & podcasts)
- Writing or journaling
- Art creation
- Hobbies such as knitting or model building
- Indoor Exercise
- Outdoor Exercise
- Computer gaming
- Non-computer games (board games or cards)
- Watching movies/series
- Engaging in social media
- New learning (online courses, webinars etc.)
- Socializing with others (through social media or phone)
- Other (please specify)
  Please enter your comment here:

Which of these activities have you engaged in with those in self-isolation with you? Please select all that apply.

- Song writing
- Listening to music
- Non-mandated reading (includes audiobooks & podcasts)
- Writing or journaling
- Art creation
- Hobbies such as knitting or model building
- Indoor Exercise
- Outdoor Exercise
- Computer gaming
- Non-computer games (board games or cards)
- Watching movies/series
- Engaging in social media
- New learning (online courses, webinars etc.)
- Socializing with others (through social media or phone)
- Other (please specify)
  Please enter your comment here:

Which of these activities have you engaged in socially using the internet? Please select all that apply.
- Song writing
- Listening to music
- Non-mandated reading (includes audiobooks & podcasts)
- Writing or journaling
- Art creation
- Hobbies such as knitting or model building
- Indoor Exercise
- Outdoor Exercise
- Computer gaming
- Non-computer games (board games or cards)
- Watching movies/series
- Engaging in social media
- New learning (online courses, webinars etc.)
- Socializing with others (through social media or phone)
- Other (please specify)
  Please enter your comment here:

Which of these activities have you spent more time doing in comparison to before March 16? Please select all that apply.
- Song writing
- Listening to music
- Non-mandated reading (includes audiobooks & podcasts)
- Writing or journaling
- Art creation
- Hobbies such as knitting or model building
- Indoor Exercise
- Outdoor Exercise
- Computer gaming
- Non-computer games (board games or cards)
- Watching movies/series
- Engaging in social media
- New learning (online courses, webinars etc.)
- Socializing with others (through social media or phone)

- Other (please specify)
  Please enter your comment here:

Which of the following do you feel have supported your overall well-being during the COVID-19 precautions? Please rate each activity from 1 (Not at all) to 7 (Extremely)

Song writing
1 2 3 4 5 6 7

Listening to music
1 2 3 4 5 6 7

Non-mandated reading (includes audiobooks & podcasts)
1 2 3 4 5 6 7

Writing or journaling
1 2 3 4 5 6 7

Art creation
1 2 3 4 5 6 7

Hobbies such as knitting or model building
1 2 3 4 5 6 7

Indoor Exercise
1 2 3 4 5 6 7

Outdoor Exercise
1 2 3 4 5 6 7

Computer gaming
1 2 3 4 5 6 7

Non-computer games (board games or cards)
1 2 3 4 5 6 7

Watching movies/series
1 2 3 4 5 6 7

Engaging in social media
1 2 3 4 5 6 7

New learning (online courses, webinars etc.)
1 2 3 4 5 6 7

Socializing with others (through social media or phone)
1 2 3 4 5 6 7

Other (please specify)
Please enter your comment here:

1 2 3 4 5 6 7

Please add any additional information that you feel confortable sharing about how you are coping at this time of change as a result of the COVID-19 pandemic.

Please comment here:

Music Background

Do you have any hearing difficulty?

- No
- Yes (please describe)

Please enter your comment here:

Do you play music professionally? If yes, please describe the situations in which you are paid to play. Please select all that apply.

- I do not play music professionally
- Performing
- Teaching
- Playing in bands or orchestra
- DJ
- Choir
- Other (please specify)

Please enter your comment here:

Can you play by ear? Playing or learning to play a piece of music by ear means you can do so by listening without the aid of printed material.

- Yes
- No

How many hours per week do you spend listening to music?

- None
- 1-10 hours
- 10-20 hours
- 20-30 hours
- 30+ hours

How closely do you pay attention when listening to music? Please rate from 1 (music is always background only) to 5 (always pay close attention to music)

1 2 3 4 5

What styles of music do you listen to?

- Rock
- R&B
- Pop
- Broadway/Musicals
- Classical
- Traditional/Folk
- Rap
- Other (please specify

Please enter your comment here:

Do any of your immediate family members play a musical instrument?
- Yes
- No

Standardized questionnaire #1: TIPI (Big 5 short version)

Standardized questionnaire #2: STAI – S

Would you be interested in participating in an online music therapy group on a drop-in basis?
- Yes
- No
- Maybe

Would you be interested in participating in an online art therapy group on a drop-in basis?
- Yes
- No
- Maybe

Would you be interested in participating in an online verbal therapy group on a drop-in basis?
- Yes
- No
- Maybe

Which of the following supports (if any) have you accessed since March 13, 2020?
- McMaster Student Wellness Centre (if comfortable, please share which supports you have accessed)
  Please enter your comment here:
- McMaster Open Circle (if comfortable, please share which supports you have accessed)
  Please enter your comment here:
- Mental Health practitioner such as: psychotherapist, psychiatrist, social worker, counsellor.
  Please enter your comment here:
- A telephone support line
  Please enter your comment here:
- Other (please specify)
  Please enter your comment here:

Thank you for your participation!

We hope that you are keeping well! Please click here for a list of wellness resources. McMaster Student Supports.

If you are interested in (1) receiving a newsletter summarizing the results of this and other studies (2) information on potential online therapy drop in groups and/or (3) participating in the draw for one of ten $50 prizes, please click the button below.
Note that this will take you to another website so that your email will not be associated with the answers you gave on this questionnaire, which will remain anonymous.

<u>B. The complete list of students' comments to the open-ended question</u> "*Please add any additional information that you feel comfortable sharing about how you are coping at this time of change as a result of the COVID-19 pandemic.*"

Positive Comments (73)

Self-Care (15)

1.Reading my old writings, going over my old drawings and thinking of the good days I can make after the pandemic is over.
2.I find myself organizing things that I've always meant to and never had the time for. Also, I find myself relaxing more and taking the time to sit down and enjoy meals.
3.By trying to maintain consistency in my day to day schedule.
4.I like to space out the time that I'm doing school work and time that I am engaging in other activities. Usually the night before, I'll try to plan at least one special thing to do so that each day seems exciting and unique!
5.I am also coping by paying more attention to my pets, as they provide me a sense of comfort.
6.Sleeping a lot, getting ready everyday such as brushing teeth and washing face, taking care of my health more than usual(taking vitamins)
7.Trying to set goals that are still reasonable to complete during the pandemic , like learn a new hobby, learning how to cook and eat healthier or trying to stay outside for multiple hours in a day.<br>Setting goals help keep me feeling optimistic and positive for the future
8.A healthy mindset, keeping one's self busy and self-awareness are key to stay positive during this tough time.
9.For me, I do my best to minimize my stress levels, and I think one of the best ways to do that is be wary of how much I use the computer and my phone. A lot of the news that is currently circulating often makes me more upset and stressed. While I do stay informed about updates on the situation, I try to minimize my usage to socializing with others and asking others how they are doing instead
10. I've enjoyed it to some degree. Lots of time to spend at home and focus on my own skills and sense of self.
11. I am just trying to take care of my self. I enjoy listening to music and socializing with my family members, who I live at home with. It is such a tough time and trying to learn something new is what I have been doing -- whether if it is a cool little fact or something new about myself as a person
12. I make sure to check in weekly with my doctor in regards to my mental health
13. For me it was important to keep a schedule. Get up, make my bed and get changed as I would regularly
14. I think attempting to realize that I am having a hard time coping has helped. by not pushing my feelings aside and actually trying to focus on them to understand that I need to do

something today besides studying to feel mentally healthier I have been getting better at coping with the negative emotions that comes with the pandemic.
15. Talking with my family about my feelings and thoughts about the pandemic, avoiding the news and virus information when not necessary (not harbouring fear)

Activities (40)

1.Cooking and learning how to make new foods is also something I've been doing.
2.I started a couple of personal projects such as a video series and a story
3.I think my environment has drastically impacted my time in isolation in a positive manner as I am able to push myself during this hard time in catching up with coursework alongside picking up new things and spending time with my family. In the past, I don't think I have spent as much family time as I am spending now amidst school and other commitments.
4.I am coping by Facetiming with my friends and my boyfriend. Me and my boyfriend watch Netflix together online at the same time by using a Google extension.
5.Been doing a lot of baking, lots of sleeping.
6.Being able to have more time for my faith has really improved my overall mood and wellness
7.Working out has helped a lot. Not only does it break up the day but it's also helped me mentally and physically feel better. Especially since social media usage has increased, body shaming and "ideal" beauty standards are more accessible and frequent than ever. So working out had made me feel like I'm making progress for myself which helps combat the negative feelings I get when scrolling through social media.
8. My parents have been teaching me how to cook quick meals and I have been meditating and learning to deal with anxiety and depression in healthy, proactive ways. I am grateful for this time to work on myself but also very worried. I feel I am taking the proper steps to create a productive second year for myself.
9. I feel great. I think it is a nice break, I have been sleeping better, eating better, and exercising more. I find peace and serenity in not having obligations of always being go go go between work, school, and other obligations. While sometimes feeling a little bored and lonely, I am staying busy and honestly quite enjoying it
10. By praying and reading my Bible. Connecting with my church. Helping to raise funds for families who have lost their jobs.
11. I've been baking a lot of bread. It makes me feel like I have control over something, also I really missed bread.
12. Meditation, cleaning
13. Spending more time with family (i.e. those who I'm self-isolating with)
14. Sleep in a lot has been really good. More time to organize school work, not in in control of the virus but in control of what I'm doing and being organized, lots of cleaning.
15. Social media engagement with and without people (virtually or in person) has been a weird way of coping that I honestly don't know if it helps me or not. It feels like I used social media to fill my time and distract myself rather than actually cope with what's going on or how I'm feeling. I definitely have also been exercising more and it provides a little bit of structure and planning to my day that I need right now since everything is at home. I think I've discovered what might work for me more when dealing with stressful factors in my life in that I used to

think singing or playing a musical instrument casually really helped me cope when things got rough. Now that I have more time at home I’ve learned that song-writer helps me way more because I’m preaching a message that is completely what I need at that moment.
16. I like making lists of things I should accomplish in a day so I don’t end up wasting all my time on social media or surfing the web (too much screen time makes me depressed)<br>My happiest moments during these times are when I can go outside and feel the sunshine (I live in rural area so we have lots of space). I love working in our gardens ☺
17. Spending more time with family and making my dad watch my favourite movie series with me as a form of bonding time
18. Self-reflection through journaling in the midst of everything truly allows me to focus better on myself, the world and see what’s important in life. Truly got to reflect on the meaning of life.
19. The only way I’m keeping sane is because I get to go out and work, otherwise my situation would be worse.
20. Keeping in contact and checking up on friends constantly
21. Since I am an international student, I am always have video call with my mom and grandparents. They already been through the self isolation, therefore they knew how I feel right now, so my mom will call me every morning and evening, I share what I did for a day, what I cook today and so on. For me try to contact with your family and your friends!
22. Cooking new recipes with music
23. Finding resources to not be behind in my plans for work, research, graduate and med applications; since limited professor accommodation has resulted from this mainly studying all day.
24. Finding time to exercise and spending time on social media has helped me focus on happier things during the pandemic.
25. Sleeping 9 hours per day
26. Making new recipes!
27. Watching the news
28. Cooking and baking is really a awesome way to kill time
29. Cooking
30. It's important to stay connected with friends and family and get some fresh air!
31. Caring for animals has helped me
32. Trying to do activities that engage my siblings and parents as well as myself to have more social interaction throughout the day. Also, trying to keep a somewhat regular daily routine has been helpful in making the days feel more normal.
33. Spending time in the outdoors
34. Since it is currently exam season, I am using more time to study as I am still in my student house. Once exams are over I feel like my habits in quarantine will change to include more exercise and hobbies.
35. Cooking more
36. According to my Nintendo Switch I have spent 95+ hours playing animal crossing and I only got it on March 31, if that gives any indication of how bored I've been. I also work full time from home (40 hrs/wk) as I am on an internship.
37. I have provided myself with a long-term goal of running a marathon that I can begin during the pandemic but can be completed even once the pandemic is over. This gives me some type of routine.

38. Honestly, I don't mind staying at home in quarantine. I have lots of things to do in the house including baking, cooking, painting, catching up with my friends through social media, and watching movies. Ever since the quarantine was announced, I have done a lot of things at home that I never had time to do in the past.
39. Reading has been a great relief to me and I am very happy to get to since I love it and don't get to very often (because of school/ work/ other commitments)
40. I am just hanging out a lot. I have done lots of cleaning and organizing and am trying to find things to keep me busy since I am someone that needs to constantly be doing something.

Positive Attitude (18)

1. I think the most helpful thing is to know that we're all in this together.
2. Doing anything to engage your entire focus so that you forget about the pandemic for a while. Realizing how many things we can still do instead of focusing on all of the restrictions.
3. Each day is a bit different, some are better and some are worse but I'm trying to stay optimistic. One really great thing about being home is that I can spend more time with family and on hobbies, but on the other hand, I really miss my friends and boyfriend.
4. I am actually doing really well in isolation. Albeit, I have my parents and a sister in the house with me, but I thrive in alone-time which is what this is. For the first week home I felt a little weird, but since then, I've been feeling really comfortable.
5. Surprisingly, my motivation for completing school work has increased since March. My grades are higher than the first semester, and the grades that I received after quarantine became are higher than other grades I received this term. I have never studied harder than I did for my final exams, and the grades displayed this. I believe this is due to being closer to my family, the environment in Hamilton was becoming a bit tedious, I wasn't eating, I had trouble sleeping, I was experiencing many anxiety attacks and depressive spells which I was not used to. Being home allowed for some familiarity and some time for healing. After exams were completed I have been taking the time to become physically active and build back the muscle I lost, I have been increasing my appetite, and I have been creating a healthy sleep schedule.
6. Just try to be positive and hopeful that all will be normal again one day.
7. I'm worried about the situation in the world, but I'm enjoying this time myself. I'm an introvert, so having more time to myself helps a lot. I have more time to focus on the things I enjoy.
8. Taking it as a mental health break, working on my health
9. One day at a time
10. Knowing that by self isolating I am not increasing the risk of anyone else getting sick.
11. I'm coping by staying positive and looking at the bigger picture, that the best thing to do is to follow the rules so we can keep everyone safe. And that this too shall end. I spend a lot of time talking to my friends who are able to share the same feelings towards the insanity of our world right now and the adjustment/ difficulty to online learning. It helps to maintain the morale to know you're not alone.
12. I have the privilege of being able to work from home so my situation has not changed drastically. For the aspects that have changed, I don't stress over things that are out of my

control. Control your immediate environment and make it somewhere you want to be for the time being.
13. Living day by day and trying to think about future big plans
14. It is always important to remember that not everyone has the same privilege of being at home with family during this pandemic. Even those that have a 'house' to be in do not necessarily have a safe 'home'. Thank you for doing this survey and gauging perceptions!
15. I'm doing pretty good, if it wasn't for school this is probably how I'd spend most of my time anyways.
16. I have been coping by staying in close contact with my friends and doing a lot of work for the MSU club I am leading along with others.
17. Once the emergency benefit money came through, I've been much more peaceful. I'm chronically ill and prefer working from home, yet without the stress of expectations to get to campus, I've been able to fully enjoy working from home and have finished my fourth year peacefully and with straight As.
18. But very thankful for Mcmaster postponing in person graduation ceremonies to a later date - it doesn't make the hard work of 4 years go to waste.

Negative Comments (78)

Negative Impact on Studies (24)

1. Teachers not giving proper help and activities make it very hard
2. It's not amazing. I definitely prefer not to live at home when it comes to my studies. Especially at a time like this where everyone is present within the house, things can get really tense. In addition, it's difficult to stick to a schedule which makes the days seem pretty long and dreary. Everything sucks lol
3. I don't understand how one could be able to sustainably learn 5 courses online for an entire year. This is a great concern for me personally heading into next year. Things have been very overwhelming lately. My thesis supervisor cancelled my placement due to COVID-19, I'm switching programs, it's my final year in university, and everything seems like it's all crashing down, and I'm feeling so helpless about everything around me.
4. Exams have been insanely anxiety provoking and stressful after having been isolated and learning online.
5. I worry that because of the financial repercussions of this pandemic, University will be a short-lived experience for myself and many others.
6. I appreciate how fair and accommodating some coordinators and departments have been through all of this (s/o to Stephen Russells, Sara Cormier, and Michelle Cadieux), but then it kind of sucks to see other departments placing restrictions on exams that seem unfair (e.g. not being able to go back to a question and change your answer). I understand that it's hard for them to ensure academic dishonesty doesn't occur, but I wish there was a better way of doing it.
7. School work is difficult to get motivated to do.

8. Lack of routine makes me restless and anxious. I can't figure out what I should be doing. With school still going any time I relax or try and do art or music I feel guilty. I'm hoping this fades as my exams end. I get upset that I spend all day on my phone (tiktok drew) and try and self regulate but lack the motivation and energy to encourage myself to do more tasks etc
9. It's kind of hard losing the routine that in-person classes gave you. Once you don't have a structure anymore, it's really up to you to keep it together. Which can be hard depending on your family life (if you stay at home/move back home), or the situations you face. I believe everyone faces hardships in one form or another, and it may get amplified during these times.
10. It is very strange to be finishing my undergrad at home. I feel like I'm in limbo a bit, with no certain end and no closure on school. I don't have a job yet.
11. Hard to do anything other than school because school takes longer to do when there are so many people around
12. Initially, was a blow to routine schedule which did not help with all the other upheaval that happened this year. Has settled into a new normal.
13. I'm not because I have so much stuff to study. Honestly, I'm not coping. I am not sure why the school keeps thinking we are. Every single professor did not feel compassion at all, and instead asked for more things to be done. Even during a pandemic, they have chosen to show us that school and education is more important than our family time and our coping time.
14. The unfortunate aspect about the COVID-19 pandemic is that it caused us to quarantine and self-isolate during a time when most of my courses were teaching material that was to be covered on exams (e.g. my CHEM 1AA3 class was going over the organic chemistry unit, which was covered extensively on the exam). Because things were so dramatically online, it disrupted a lot of learning that was important. Additionally, I personally felt that moving out of residence and going back home was very stressful and challenging, both on my academics and on my mental well-being. A lot of us, myself included, don't have proper access to internet or a quiet space to study and work. Living on residence was what allowed me to really focus on myself and my work, and I was not happy that the university decided to make us move out especially at a time when exam preparations were underway. Additionally, the financial costs associated with this pandemic have been pretty stressful too.
15. School has been really stressful. The shift to online tests has resulted in a mark drop for me because I've felt that the rests are now harder. Also having more trouble focusing and studying and being productive. Constantly feeling tired, just want to sleep. And rest.
16. Just finding things to occupy myself with around the house and searching random stuff on the internet. Also trying to use this time to plan my future, as I am stressed that no standardized tests for DAT or OAT or MCAT are taking place, which I had planned to study for and write this summer.
17. I found it extremely difficult to keep up with assignments throughout March and the beginning of April. It was also difficult not being able to study in a quiet environment or at the library.
18. Really badly, I couldn't cope because of the sudden changes and so many things have been shifted in my life that I couldn't even keep with school and failed academically. I ended up being more stressed, depressed and hopeless.
19. Some of my professors at McMaster have been absolutely atrocious and extremely unaccommodating with all that is in mind leading to deteriorating mental health and

	unnecessary struggling. On the other hand, some professors have been showing a copious amount of understanding and empathy while maintaining an adequate and not too lenient system for science courses.
20.	I definetly feel that profs needed to work out the marking scheme in a better way. Many Universities offered the option of mark freezing, which would have been extremely helpful
21.	My home environment is not exactly suitable for 10h studying a day for exams, so professors needed to have supported students and understand the mark on their exams may not reflect the student. Exams have been insanely anxiety provoking and stressful after having been isolated and learning online.
22.	online school sucks, I usually have a lot of motivation to study for exams, but I feel drained constantly and am always distracted
23.	a lot of wasted time on social media, all my time goes to studying for exams
24.	studying is harder as home is not a library, but oh well

Negative Impact on Well-being (45)

1.	Schoolwork is difficult to get motivated to do, also not having an ideal living situation severely impacts how isolation is going
2.	I find it very hard to be motivated to do essentially anything. I stay in bed until 1 every day because I know that the day will be boring when i eventually get up.
3.	I've been coping alright, but I've lost a lot of motivation. It usually comes in waves.
4.	My social media use has dramatically increased since I'm not occupied by attending lectures or visiting the library to study. As a result, my procrastination has also increased dramatically. This honestly causes me a lot of anxiety because I've been running out of time and haven't been able to put my normal effort into my studies (which is still my fault, but when you're trapped inside, it's difficult to ignore the freedom that the internet offers).
5.	I've been sleeping a lot more often than I usually would. Honestly feel like the only reason I do that is to make the days pass quicker.
6.	The pandemic really affected my motivation level and mood. Even though we have a lot more time to get work done, it is a lot harder to get the motivation to actually sit down and be productive. Moreover, it really impacted my sleep schedule.
7.	I believe anti-depressants are probably helping a lot in addition to the self-care and exercise I have been participating in as I am coping. However, the changes to the world/lifestyle and anxieties about the pandemic have seemed to worsen psychological symptoms.
8.	Hard to cope, tougher if you have mild depression, studying is harder as home is not a library, but oh well
9.	Phone calls with therapist
10.	I'm having a really difficult time. Living with my mental illness is so much more difficult in the present circumstances. I know were all in this "together" but it doesn't feel like that when you're separated from the people who support you and all of your coping strategies are either stretched thin or unavailable.
11.	Loss of personal connection, last year of undergraduate completely changed.

12. taking lorazepam
13. Everything is so awful right now. I already have depression that makes it extremely difficult to do my work and now I am still expected to function at a high level? I wasn’t functioning before the pandemic.
14. The pandemic has caused me to feel extremely trapped and hopeless for the future. I struggle with my mental health and the current situation has caused me to regress to a state that took 6 years of intense psychotherapy and pharmaceutical intervention to manage. Mundane tasks, such as picking up my prescriptions or visiting the doctor, have become increasingly difficult and impossible. The lack of a definitive endpoint for all of this has caused me the most stress. I recognize that this situation has been difficult for everyone and I hope that the government, large corporations, and academic institutions will approach post-COVID-19 life with compassion, patience, and generosity
15. Not coping at all
16. Nothing it sucks and makes my depression awful
17. It is particularly hard not seeing my friends from school every day. It was hard knowing I didn’t get to wish them the best…even though we are keeping in touch virtually.
18. I work full time as I am on internship. I am currently working from home and that takes up most of my weekdays. I also play a lot of animal crossing, go on Tiktok (which I only downloaded after quarantine as a desperate measure to combat my boredom) and FaceTiming loved ones
19. I am doing alright – as an introverted person, life has not changed too much for me. But I hope that this can all end soon, because as much as I do enjoy spending time alone, I really do miss my school friends.
20. Difficult to be at home due to family issues, hard to be away from friends and other loved ones
21. At times, I feel quite lonely from staying at home for so long.
22. Working full time keeps you busy, without that I would be bored at home.
23. I am very bad at communication using a phone and prefer in person interaction and communication, so it has been difficult for me to adjust in that way.
24. One really great thing about being home is that I can spend more time with family and on hobbies, but on the other hand, I really miss my friends and boyfriend.
25. According to my Nintendo Switch I have spent 95+ hours playing animal crossing and I only got it on March 31, if that gives any indication of how bored I’ve been. I also work full time from home (40 hrs/wk) as I am on an internship.
26. I feel a constant sense of worry about how coronavirus will affect the people around me and my future. I also miss seeing my friends and family in real life and being able to do activities with them.
27. I am bored and have a lot of time to just sit and think. I don’t get sad, it’s just hard to sleep sometimes.
28. I basically masturbate 2000 times a day which is pretty much toxic. Being lonely literally drives me sad and I realized that life is actually fairly short!!!
29. I live in a house with other people but I have separate entrance and do not know them, so have been feeling very lonely especially when I see other people spending time and doing activities with roommates or housemates. I usually enjoy living alone and it

works for me but being self-isolated by myself has been difficult as I have no in person interaction anymore, only online.
30. Social media engagement with and without people (virtually or in person) has been a weird way of coping that I honestly don’t know if it helps me or not. It feels like I used social media to fill my time and distract myself rather than actually cope with what’s going on or how I’m feeling.
31. I will sometimes just call a friend and leave them on a video call, buy we won’t talk much. I have to live in student housing still because my wifi at home is not strong enough to support our whole family using it. It gets very lonely, so video calls are necessary for me to stay well.
32. It’s a very difficult time to not live with my partner. Especially when they are a front line worker and I am stuck at home worried.
33. I am super fortunate that my boyfriend and I moved in together in February so we ended up being in isolation together otherwise I don’t know if we would be seeing each other. I do miss my family and I truly appreciate talking on the phone with them.
34. The abundance of sleep and the lost sense of time adds to the lack of reality and realization of current events.
35. Just worried about how much longer we have to wait during this pandemic.
36. My social media use has dramatically increased since I’m not occupied by attending lectures or visiting the library to study. As a result, my procrastination has also increased dramatically. This honestly causes me a lot of anxiety because I’ve been running out of time and haven’t been able to put my normal effort into my studies (which is still my fault, but when you’re trapped inside, it’s difficult to ignore the freedom that the internet offers).
37. I feel a constant sense of worry about how coronavirus will affect the people around me and my future. I also miss seeing my friends and family in real life and being able to do activities with them.
38. Just stressed out about the fall term will look and if I will be able to work this summer.
39. Exams have been insanely anxiety provoking and stressful after having been isolated and learning online.
40. It’s a very difficult time to not live with my partner. Especially when they are a front line worker and I am stuck at home worried.
41. This has severely affected both my mental and physical health. I need exercise (gym) to manage my mood and I have not been able to work out the way I like throughout this pandemic. In my free time I enjoy seeing live music with my friends but I have not been able to do that since the first week of March. I have now finished exams but I am not able to work due to the pandemic. I am currently living at home with my family, but I still have an apartment in Hamilton that I am responsible for. The recent announcement about funding for students will not cover my schooling, rent, utilities, food, etc. The money that I make during the summer has to last me through the entire school year. As the government funding is much less than what I usually make per month in the summer (I have been working at the same job for 4 years and I have been promoted/given a raise) I am extremely concerned about my financial situation in the fall. Additionally, I am concerned that I will not be able to take certain required courses in the fall (experimental learning/community engagement courses) and I will have to defer my graduation to the following academic year.

42.	I want to go to the grocery store without fear
43.	Lack of routine makes me restless and anxious.
44.	It's been stressful and boring but we all have to do our part for this to be over :)
45.	It has been extremely difficult not being able to go outside but I am trying my best to cope especially with financial strains.

Negative Due to Living Arrangements (9)

1.	Also not having an ideal living situation severely impacts how isolation is going
2.	Difficult to be at home due to family issues, hard to be away from friends and other loved ones
3.	Due to COVID, my parents got me to move back in with them. I love them, but living with them definitely has a negative impact on my wellness.
4.	It is always important to remember that not everyone has the same privilege of being at home with family during this pandemic. Even those that have a ‘house’ to be in do not necessarily have a safe ‘home’. Thank you for doing this survey and gauging perceptions! I am feeling quite restless at home and it is difficult to stay productive here.
5.	I am not coping well, being in forced close proximity with my family and previously my roommates has caused my relationships with them to shift. My mental health has suffered a sharp decline.
6.	Being isolated with one person this whole time has caused my mental health to deteriorate so rapidly, I don’t know if I can come out of this sane.
7.	It has been stressful, always being home, getting in each other’s way… generally, my family tries to keep out of each other’s way. When we come together, we enjoy each other’s company, but we don’t push it.
8.	I live in a house with other people but I have separate entrance and do not know them, so have been feeling very lonely especially when I see other people spending time and doing activities with roommates or housemates. I usually enjoy living alone and it works for me but being self-isolated by myself has been difficult as I have no in person interaction anymore, only online.
9.	I will sometimes just call a friend and leave them on a video call, buy we won't talk much. I have to live in student housing still because my wifi at home is not strong enough to support our whole family using it. It gets very lonely, so video calls are necessary for me to stay well.
